# Partial cardiopulmonary bypass through left thoracotomy for coarctation repair in children

**DOI:** 10.1186/s13019-024-02849-x

**Published:** 2024-06-22

**Authors:** Kunihiko Joo, Yoshie Ochiai, Yuma Motomatsu, Yuki Hashizumi, Yutaka Maniwa, Yuichiro Sugitani, Mamie Watanabe, Jun Muneuchi, Shigehiko Tokunaga

**Affiliations:** 1 Department of Cardiovascular Surgery, JCHO Kyushu Hospital, 1-8-1 Kishinoura, Yahata-nishi-ku, Kitakyushu City, 806-8501 Japan; 2 Department of Pediatric Cardiology, JCHO Kyushu Hospital, Kitakyushu City, Japan

**Keywords:** Coarctation of the aorta, Left thoracotomy, Partial cardiopulmonary bypass

## Abstract

**Background:**

A left thoracotomy approach is anatomically appropriate for childhood aortic coarctation; however, the pediatric femoral arteriovenous diameters are too small for cardiopulmonary bypass cannulation. We aimed to determine the safety of a partial cardiopulmonary bypass through the main pulmonary artery and the descending aorta in pediatric aortic coarctation repair.

**Methods:**

We retrospectively reviewed 10 patients who underwent coarctation repair under partial main pulmonary artery-to-descending aorta cardiopulmonary bypass with a left thoracotomy as the CPB group. During the same period, 16 cases of simple coarctation of the aorta repair, with end-to-end anastomosis through a left thoracotomy without partial CPB assistance, were included as the non-CPB group to evaluate the impact of partial CPB.

**Results:**

The median age and weight at surgery of the CPB group were 3.1 years (range, 9 days to 17.9 years) and 14.0 (range, 2.8–40.7) kg, respectively. Indications for the partial cardiopulmonary bypass with overlap were as follows: age > 1 year (*n* = 7), mild aortic coarctation (*n* = 3), and predicted ischemic time > 30 min (*n* = 5). Coarctation repair using autologous tissue was performed in seven cases and graft replacement in three. The mean partial cardiopulmonary bypass time, descending aortic clamp time, and cardiopulmonary bypass flow rate were 73 ± 37 min, 57 ± 27 min, and 1.6 ± 0.2 L/min/m^2^, respectively. Urine output during descending aortic clamping was observed in most cases in the CPB group (mean: 9.1 ± 7.9 mL/kg/h), and the total intraoperative urine output was 3.2 ± 2.7 mL/kg/h and 1.2 ± 1.5 mL/kg/h in the CPB and non-CPB group, respectively (*p* = 0.020). The median ventilation time was 1 day (range, 0–15), and the intensive care unit stay duration was 4 days (range, 1–16) with no surgical deaths. No major complications, including paraplegia or recurrent coarctation, occurred postoperatively during a median observation period of 8.1 (range, 3.4–17.5) years in the CPB group. In contrast, reoperation with recurrent coarctation was observed in 2 cases in the non-CPB group (*p* = 0.37).

**Conclusions:**

Partial cardiopulmonary bypass through the main pulmonary artery and descending aorta via a left thoracotomy is a safe and useful option for aortic coarctation repair in children.

## Background

A left lateral thoracotomy is often preferred over a median sternotomy for repairing coarctation of the aorta in childhood due to its ability to create a wider subaortic space through the extended end-to-end anastomosis (EEA) procedure [1]. Typically, most repairs for coarctation of the aorta via left thoracotomy in children are performed using a simple aortic cross-clamp. Paraplegia, while rare, remains a potential complication of this procedure [2], with devastating consequences and a significant impact on patient quality of life, thus necessitating its prevention. To mitigate paraplegia risk, a partial cardiopulmonary bypass (CPB) is recommended, especially for older children or those with inadequate collaterals to the lower body [3]. However, while a partial CPB is commonly established in adults through femoral arteriovenous cannulation, a majority of children possess an inadequate femoral arteriovenous vascular diameter for cannulation. In this study, we analyzed surgical outcomes of main pulmonary artery-to-descending aorta partial CPB through a left thoracotomy performed for circulatory support during the coarctation of the aorta repair. We aimed to verify the safety of this procedure in children.

## Methods

### Patient characteristics and procedure

**Fig. 1:**
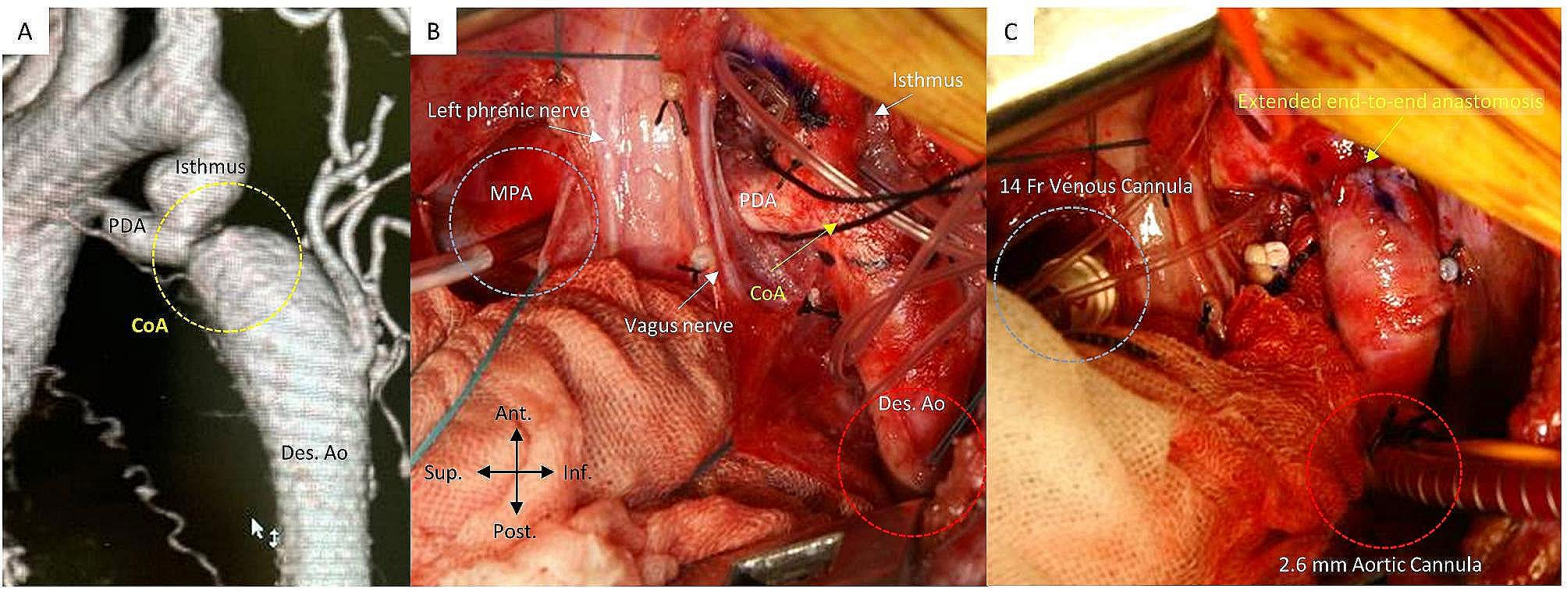
Preoperative imaging and intraoperative findings in Case #8. **A**: Preoperative CT findings. **B**: Intraoperative findings before initiating the main pulmonary artery-to-descending aorta partial cardiopulmonary bypass. **C**: Intraoperative findings after CoA repair by extended end-to-end anastomosis (CoA, coarctation of the aorta; CT, computed tomography; Des.Ao, descending aorta; PDA, patent ductus arteriosus.)

This retrospective study was approved by the Institutional Ethics Committee of the JCHO Kyushu Hospital (Fukuoka, Japan). We considered the following as indications for partial CPB requiring coarctation of the aorta repair: (1) age > 1 year, (2) mild coarctation of the aorta without adequate collateral, and (3) predicted descending aortic clamp time of > 30 min, such as graft replacement or reoperation for recurrent coarctation of the aorta. Aortic arch morphology with anatomical features of coarctation but with a pressure gradient of < 20 mmHg was defined as mild coarctation of the aorta. Between January 2006 and July 2023, 10 patients underwent a coarctation of the aorta repair through the left thoracotomy using a main pulmonary artery-to-descending aorta partial CPB (the CPB group); this group comprised eight boys and two girls (both with Turner syndrome). During the coarctation of the aorta repair, the median age was 3.1 years (range: 9 days–17.9 years), and the median weight was 14.0 kg (range: 2.8–40.7). Seven patients had bicuspid aortic valves, whereas one had concomitant mitral valve stenosis secondary to the Shone complex. No patient had a comorbid aberrant right subclavian artery. All patients underwent preoperative computed tomography for detailed anatomical analyses of the aortic arch (Fig. 1A) following echocardiography. Nine patients underwent preoperative catheter examination to evaluate the coarctation of the aorta pressure gradient and left ventricular function. In addition, the development of collateral blood circulation to the lower body was evaluated based on computed tomography and catheter imaging data. These preoperative evaluations were reviewed at a conference to determine the appropriate surgical procedures. Sixteen cases of simple coarctation of the aorta repair with EEA or extended EEA performed through a left thoracotomy without partial CPB assistance during the same period were included as the non-CPB group to evaluate the effect of partial CPB on the lower body organs and the incidence of its associated complications. The non-CPB group included two infants older than 1 year (2.2 and 1.0 years old, respectively), both of whom were amongst the earliest cases in the current study. All patients underwent postoperative computed tomography or cardiac catheter examination during hospitalization. Blood pressure measurements of the upper and lower extremities and echocardiography were performed in the outpatient clinic after discharge. Surgical and CPB data, anesthesia records, and the postoperative course were retrospectively examined.

### Surgical technique

After inducing general anesthesia, patients were monitored by placing arterial lines in their right radial arteries and dorsal or posterior tibial arteries as much as possible. All patients were placed in the right supine position and were approached through the third or fourth intercostal space during the left posterolateral thoracotomy. The descending aorta was taped and dissected distally to at least three intercostal spaces. The intercostal arteries were preserved as much as possible, and the lymphatic capillaries were ligated. The transverse arch was dissected on the proximal side, and the left subclavian and left carotid arteries were taped. The ductus arteriosus was divided after ligation, carefully avoiding damage to a recurrent nerve. The distal aortic arch provided passivity to the descending aorta. The pericardium was then opened longitudinally anterior to the left phrenic nerve, and the main pulmonary artery trunk was taped. The patient was systematically heparinized after a purse-string suturing of the distal descending aorta and the main pulmonary artery just above the Valsalva (Fig. 1B). After confirming that the activated clotting time was extended to > 400 s, cannulations were performed on the descending aorta using a right-angle cannula and on the main pulmonary artery using a straight cannula (DLP; Medtronic INC, Minneapolis, Minnesota); a partial CPB was then initiated. Ventilation was continued at a reduced volume while monitoring oxygen saturation of the right hand, and one-lung ventilation was not performed. The CPB flow was targeted at approximately 50% of the calculated maximum flow of 2.8–3.2 L/min/m^2^, while maintaining a normal right radial artery pressure and lower blood mean pressure > 40 mmHg. Medications for blood pressure control during the assisted circulation were primarily inotropic agents and chlorpromazine. The rectal temperature was maintained in the low range at 35.0 °C during descending aortic clamping. The coarctation of the aorta tissue was fully resected after clamping the descending aorta and the distal aortic arch; the aortic arch was then reconstructed using extended EEA with or without autologous tissue or artificial graft replacement (Fig. 1C). The aortic clamps were released after adequate air evacuation. Heparin neutralization with protamine was promptly performed, and the cannulas were removed. The pericardium was roughly closed after carefully checking for bleeding or lymphatic leakage around the descending aorta. One drain was placed in the left thoracic cavity, and the chest was closed. An almost identical surgical procedure was used for coarctation of the aorta repair without partial CPB support.

### Statistical analysis

Means and standard deviations have been used to present the distribution of the continuous dataset. Continuous and categorical variables were compared between the cardiopulmonary and non-CPB groups using the Student’s *t*-test and the one-sided Fisher’s exact test, respectively. Statistical significance was set at *p* < 0.05. Statistical analyses were performed using GraphPad Prism version 5 (GraphPad Prism Software Inc., San Diego, CA, USA).

## Results

Coarctation of the aorta resection and extended EEA were performed in seven cases, with two cases involving patch augmentation using 0.6% glutaraldehyde-treated autologous pericardium. In three cases, graft interposition was necessary, with 18 mm vascular grafts used in two cases (J-graft; Japan Lifeline, Tokyo, Japan) and a 16-mm vascular graft used in one (Hemashield Platinum; Maquet, Rastatt, Germany). Seven of the patients were aged > 1 year, including two with mild coarctation of the aorta and three with graft interposition. Another three cases (cases #4, #9, and #10) were aged < 1 year. Case #4 involved a patient with the Shone complex, who was likely to later require surgical intervention with median sternotomy for a left ventricular outflow obstructive lesion; therefore, only a mild coarctation of the aorta repair was performed using left thoracotomy. Initially, the coarctation of the aorta was repaired using extended EEA with the preservation of the aberrant left subclavian artery branching near the ductus arteriosus. However, there was still a residual coarctation of the aorta with a pressure gradient of 30 mmHg, and anastomosis was repeated with the scarification of the left subclavian artery (total aortic clamp time: 49 + 49 min). Cases #9 and #10 included infants weighing 3.2 and 3.7 kg, respectively, with stenosis at the site of extended EEA reconstruction that required reoperation within the same hospitalization period. Both patients exhibited coarctation with long tubular isthmus, prompting a left thoracotomy approach for the initial repair. Due to the distance between the descending aorta and aortic arch, tension was placed on the anastomosis, leading to anastomotic stenosis. Consequently, the patients underwent patch augmentation at the lesser curvature side of the extended EEA with a 0.6% glutaraldehyde-treated fan-shaped autologous pericardium with partial CPB because the predicted aortic clamp time exceeded 30 min. The patients’ demographic characteristics are summarized in Table 1.

**Table 1:** Patient characteristics in the CPB group

Patient number	Age (y: years, d: days)	Body weight (kg)	Other anomalies	Pressure gradient (mmHg)	Indication for partial CPB	Surgical procedure
1	17.9 y	39.5	BAV, Turner syndrome	64	Age, graft replacement	Graft replacement (Hemashield #16)
2	2.7 y	14.8	BAV, PFO	20	Age, mild CoA	Extended EEA
3	4.1 y	13.2	Turner syndrome	21	Age, mild CoA	Extended EEA
4	9 d	2.8	MS, PDA, aberrant LSCA Shone complex	15	Mild CoA	Extended EEA
5	10.1 y	28.9	None	35	Age, graft replacement	Graft replacement (J-graft #18)
6	3.5 y	14.9	BAV, mild SAVS	50	Age	Extended EEA
7	12.7 y	40.7	BAV	58	Age, graft replacement	Graft replacement (J-graft #18)
8	1.2 y	7.9	BAV, PDA, MR	37	Age	Extended EEA
9	20 d	3.2	BAV, MS, PFO, PDA	20	Reoperation	Extended EEA with AP patch
10	62 d	3.7	PFO	40	Reoperation	Extended EEA with AP patch

AP, autologous pericardium; BAV, bicuspid aortic valve; CoA, coarctation of the aorta; CPB, cardiopulmonary bypass; EEA, end-to-end anastomosis; LSCA, left subclavian artery; MR, mitral regurgitation; MS, mitral valve stenosis; PDA, patent ductus arteriosus; PFO, patent foramen ovale; SAVS, supravalvular aortic stenosis

The cannula size used for partial CPB was selected based on the body surface area. The mean CPB time was 73 ± 37 (range: 37–130) min, and the mean descending aortic clamp time was 57 ± 27 (range: 29–98) min. During descending aortic clamping, the mean CPB flow was 1.6 ± 0.2 (range: 1.3–2.0) L/min/m^2^ and the urine output was 9.1 ± 7.9 mL/kg/h. Two patients (cases #4 and #9) were anuric intraoperatively; however, both were infants with obstruction at the tip of the urinary catheter, and urination was observed immediately after intensive care unit admission (Table 2).

**Table 2:** Results of the main pulmonary artery-to-descending aorta partial cardiopulmonary bypass

Patient number	BSA (m^2^)	Aortic cannula (mm)	MPA cannula (Fr)	CPB time (min)	Clamp time (min)	Mean CPB flow* (L/min/m^2^)	Distal mBP* (mmHg)	Urine* (mL/kg/h)	Maximum blood lactate (mmol/L)
1	1.28	4.0	24	70	62	-	-	17.1	-
2	0.60	3.0	18	38	29	1.67	56	18.1	2.2
3	0.56	3.0	16	38	32	1.68	-	4.3	2.0
4	0.18	2.0	12	62 + 70	49 + 49	1.63	40	0.0	3.4
5	1.02	3.5	20	86	76	1.30	52	7.4	2.7
6	0.63	3.0	16	37	28	1.58	-	8.1	1.3
7	1.30	3.5	22	61	50	1.51	-	23.3	3.1
8	0.39	2.6	14	39	25	1.97	49	3.7	1.2
9	0.19	2.0	12	109	87	1.47	53	0.2	1.7
10	0.23	2.4	12	120	78	1.60	41	8.6	1.0
Mean ± SD				73 ± 37	57 ± 27	1.6 ± 0.2	49 ± 6.5	9.1 ± 7.9	2.1 ± 0.9

BSA, body surface area; CPB, cardiopulmonary bypass; mBP, mean blood pressure; MPA, main pulmonary artery; SD, standard deviation; -, lack of data; *, during cardiopulmonary bypass

Individuals in the CPB group were older and had more weight because partial CPB is indicated in patients aged > 1 year. The mean descending aortic clamp time in the non-CPB group was 27.3 ± 9.3 min (*p* < 0.001). Six patients in the CPB group had a descending aortic clamp time of > 40 min. Conversely, the urine output during surgery was 3.2 ± 2.7 mL/kg/h in the CPB group and 1.2 ± 1.5 mL/kg/h in the non-CPB group (*p* = 0.020), with no increase in the postoperative serum creatinine levels.

No postoperative deaths, postoperative paraplegia, left recurrent nerve palsy, left phrenic nerve injury, or systemic embolic or hemorrhagic events occurred in either group. In the non-CPB group, chylothorax occurred in two patients; both were not administered medical therapy and were treated using thoracic duct ligation (one on postoperative day 7 and the other on postoperative day 14). In addition, early postoperative re-coarctation of the aorta was observed in two patients in the non-CPB group; percutaneous balloon angioplasty was performed in one patient at 1 month postoperatively and in the other patient at 10 months postoperatively. In case #4, reoperation with a modified Konno procedure was performed 2.2 years after coarctation of the aorta repair because of the progression of subaortic stenosis. This was followed by the Ross–Konno procedure and mitral valve repair 8.7 years after the initial surgery. No cases of late mortality were observed during the median observation period of 8.1 years (range, 0.04–17.5) (Table 3).

**Table 3:** Comparison of the perioperative outcomes between the CPB and non-CPB groups

	CPB (*n* = 10)	Non-CPB (*n* = 16)	*p*-value
Age (years)	3.1 (0.03–17.9)	0.13 (0.02–2.2)	0.004
Body weight (kg)	14.0 (2.8–40.7)	3.4 (2.0–9.1)	0.002
Operation time (min)	263 ± 78	145 ± 42	< 0.001
Descending aortic clamp time (min)	56.5 ± 27.3	27.3 ± 9.3	< 0.001
Minimum rectal temperature (°C)	34.8 ± 0.6	35.0 ± 0.8	0.67
Intraoperative urine output (mL/kg/h)	3.2 ± 2.6	1.2 ± 1.5	0.02
Preoperative serum creatinine (mg/dL)	0.41 ± 0.16	0.37 ± 0.20	0.61
Postoperative maximum serum creatinine (mg/dL)	0.49 ± 0.27	0.45 ± 0.37	0.68
Ventilation time (day)	1 (0–15)	1 (0–4)	0.45
ICU stay (day)	2 (1–16)	5 (2–10)	0.86
Allogeneic blood transfusion	4	11	0.15
Paraplegia	0	0	1
Systemic embolism	0	0	1
Bleeding event	0	0	1
Chylothorax	0	2	0.37
Re-CoA	0	2	0.37
Peak velocity at latest echocardiography (m/s)	2.1 (1.3–3.0)	2.0 (1.3–2.6)	0.42
Late mortality	0	0	1
Follow-up period (year)	8.1 (3.4–17.5)	8.3 (0.04–15.0)	0.99

CPB, cardiopulmonary bypass; ICU, intensive care unit; Re-CoA, recurrent coarctation of the aorta

## Discussion

Coarctation of the aorta manifests as the narrowing of stenosis, which occurs due to ductus arteriosus closure. This closure causes increased left ventricular afterload and decreased lower body circulation. While patients are often diagnosed in the neonatal period and promptly undergo treatment with surgical repair, delays in diagnosis may occur, albeit rarely. For instances, delays may occur if thestenosis is moderate or severe but progresses slowly, and if sufficient collateral vessels have developed [4]. The approach for coarctation of the aorta repair in neonates is controversial, particularly if the transverse arch is hypoplastic [5]. Furthermore, EEA, extended EEA, end-to-side aortoplasty, or subclavian aortoplasty is performed through either a left thoracotomy or median sternotomy, depending on the aortic arch anatomy and the policy of the individual institution. The extended EEA procedure through a left lateral thoracotomy may be optimal in older children due to two reasons: (1) many patients have localized coarctation of the aorta without the hypoplasia of the transverse arch, and (2) the lack of tissue extensibility compared with that in neonates requires dissection to the distal side of the descending aorta.

The risk factors for paraplegia following a simple aortic clamp include the cross-clamp time [6], body temperature [7], distal aortic pressure [8], and an aberrant right subclavian artery [6]. There have been various reports of acceptable limits for these parameters, but definitive cutoff values have not been identified. Regardless of the surgical technique, prolonged aortic clamp time due to intraoperative problems (such as tear, residual gradient, change of planned procedure) could occur. Therefore, it is necessary to develop a safe partial CPB technique as a bail-out strategy. The femoral artery and vein are commonly used in adult patients as access vessels for partial CPB. However, careful judgment is required in pediatric patients because vascular injury during cannulation with spasms may occur, even with an adequate femoral artery-vein diameter [9]. In addition, there are concerns regarding complications, such as infections, lymph fistulas, and neuropathy, due to the creation of a new incision in the groin.

A left heart bypass through the left atrial appendage and the descending aorta is an excellent alternative to CPB under a left thoracotomy [1, 3]. Unfortunately, the left atrial wall in children is very fragile, making cerebral complications from air embolization associated with left atrial cannulation potential risks [3]. In addition, a left heart bypass without an oxygenator does not solve lung problems associated with ventilation during the coarctation of the aorta repair. Sandrio and colleagues reported on 15 patients aged 7–48 years, with a minimum weight of 19.7 kg, who underwent partial CPB through a left thoracotomy using alternative cannulation sites (namely, main pulmonary artery and descending aorta), with no instances of paraplegia observed [10]. In this study, where we extended this technique to younger infants, we also chose the main pulmonary artery as the venous cannulation site for the following reasons. One, the vessel wall of the main pulmonary artery is more solid than that of the left atrium tissue. Two, since the venous cannulation site is in the right heart system, the possibility of air embolization into the systemic circulation is extremely low. Finally, because blood is drawn from the pulmonary artery, excessive blood supply to the ventilated right lung is suppressed, potentially reducing pulmonary complications, such as pulmonary congestion or hemorrhage. We achieved a stable targeted flow rate during assisted circulation in infants by using a straight cannula and ensuring its tip remained in the main pulmonary artery.

There is no absolute preoperative evaluation method for determining whether the collateral blood supply to the lower body is adequate, especially if the degree of coarctation is moderate. Additionally, determining the necessity of partial CPB is difficult. The intraoperative descending aortic clamp test, to measure the perfusion pressure, has been reported as a potential evaluation method [8, 11]. However, it still lacks reliability and reproducibility, as it may be influenced by the peripheral circulation status under general anesthesia with mild hypothermia. Particularly in older children, poor elasticity of vascular tissue or unexpected bleeding associated with a higher blood pressure than that of neonates may necessitate a change in surgical procedure or a prolonged aortic clamp time. Further, rectal temperature regulation with blankets is challenging in older children and can lead to uncontrolled hyperthermia [11]. The main pulmonary artery-to-descending aorta partial bypass allows reliable assisted circulation and lower body thermoregulation with fewer complications and without wound formation. This considerably lowers the threshold for the decision to use partial CPB for coarctation of the aorta repair.

Coarctation of the aorta repair through a median sternotomy with total body perfusion is another method for preventing lower body ischemia and may be preferable when other intracardiac comorbidities are present [12]. However, studies have shown balanced growth of the aortic arch with extended EEA repair via left thoracotomy [3, 13]. Utilizing a safer method of partial CPB, such as main pulmonary artery-to-descending aorta bypass, would expand the indications for more complex aortic arch repairs through a left thoracotomy. We extended the indications for main pulmonary artery-to-descending aorta partial CPB to infants weighing approximately 3 kg. Two of our infant patients needed reoperations for re-coarctation of the aorta (cases #9 and #10), and another (case #4) underwent a second aortic clamping for residual coarctation of the aorta. The prolonged aortic clamp times in these cases allowed us to perform a complex aortic arch reconstruction procedure. Even in infants, the cannulas inserted in the descending aorta and main pulmonary artery did not interfere with the surgical field during the left thoracotomy. In addition, we obtained good results using partial CPB during graft replacement, for a distal tortuous thoracic aortic aneurysm in a 1-year-old girl weighing 12.3 kg and with PHACE syndrome [14].

This study has some limitations. First, this was a retrospective study involving a small number of patients. Second, the background of the patients in the non-CPB group did not align with our indications for using a partial CPB, and we were unable to rigorously examine the protective effects on the lower body organs.

## Conclusion

We successfully performed a coarctation repair through a left thoracotomy with a partial CPB, between the main pulmonary artery and the descending aorta in 10 children. A main pulmonary artery-to-descending aorta partial CPB protects against spinal cord ischemia, even with prolonged aortic clamping. However, designing a randomized trial with or without the use of partial CPB for children at a risk of paraplegia is difficult. As a precautionary measure, surgeons should be prepared to consider the use of CPB assistance as an alternative method in all cases of coarctation of the aorta repair. Further investigations are needed; however, this method is a safe and useful option for aortic arch repair in children at a risk of paraplegia.

## Data Availability

No datasets were generated or analysed during the current study.

## Abbreviations

AP: Autologous pericardium
BAV: Bicuspid aortic valve
CoA: Coarctation of the aorta
CPB: Cardiopulmonary bypass
EEA: End-to-end anastomosis
LSCA: Left subclavian artery
MR: Mitral regurgitation
MS: Mitral valve stenosis
PDA: Patent ductus arteriosus
PFO: Patent foramen ovale
Re-CoA: Recurrent coarctation of the aorta
SAVS: Supravalvular aortic stenosis
